# Comparable Effectiveness of Novel and Commercial Saliva Substitute Gels in Dental Patients Experiencing Xerostomia: A Randomized, Double-Blind Crossover Trial

**DOI:** 10.3390/gels12010061

**Published:** 2026-01-08

**Authors:** Supanee Thanakun, Wipaporn Kajornwongwattana, Boonruthai Wattanaurai, Chanchanan Kobutr, Chayapa Parnnium, Kankanit Konta, Pornpailin Vasusopon, Supitchaya Lomloy, Thanapat Songsak, Suchada Vuddhakanok

**Affiliations:** 1 Division of Oral Diagnostic Science, College of Dental Medicine, Rangsit University, Pathum Thani 12000, Thailand; 2 Department of Pharmacology, Faculty of Medicine, Kasetsart University, Bangkok 10900, Thailand; wipaporn.kaj@ku.ac.th; 3 College of Dental Medicine, Rangsit University, Pathum Thani 12000, Thailand; boonruthai.w63@rsu.ac.th (B.W.); chanchanan.k63@rsu.ac.th (C.K.); chayapa.p63@rsu.ac.th (C.P.); kankanit.k63@rsu.ac.th (K.K.); pornpailin.v63@rsu.ac.th (P.V.); supitchaya.l63@rsu.ac.th (S.L.); suchada.v@rsu.ac.th (S.V.); 4 Department of Pharmacognosy, College of Pharmacy, Rangsit University, Pathum Thani 12000, Thailand; thanapat.s@rsu.ac.th

**Keywords:** dental application, oral disease prevention, oral wetness, salivary substitute, xerostomia

## Abstract

Saliva substitutes are the standard treatment for dry mouth. This study aimed to evaluate the clinical effectiveness of a novel artificial saliva gel (RSU gel) compared with a commercial product (GC Dry Mouth Gel^®^). A randomized, double-blind, two-phase crossover clinical trial was conducted with 37 participants with xerostomia. In the short-term phase, oral wetness, xerostomia scores, and clinical score of oral dryness (CSOD) were assessed up to 60 min after a single gel application. In the short-term repeated-use phase, each gel was applied 4 times daily for 14 days, separated by a 14-day washout period. The same parameters, including patient satisfaction and adverse events, were re-evaluated. Data were analyzed using generalized linear mixed models and generalized estimating equations. Both the RSU and GC Dry Mouth Gel^®^ significantly improved oral wetness immediately after a single application. No significant difference was observed for the RSU gel relative to the GC Dry Mouth Gel^®^ for oral wetness (OR = 1.01, 95% CI 0.98, 1.04, *p* = 0.248), xerostomia score (OR = 1.10, 95% CI 0.42, 2.88, *p* = 0.661), or CSOD (OR = 0.95, 95% CI 0.58, 1.55, *p* = 0.765) at 60 min. After 14 days of use, oral wetness increased significantly in both groups (2.94%, 95% CI 0.30%, 5.76%, *p* = 0.030) and did not differ significantly between the two products (*p* = 0.110). The xerostomia scores and CSOD also significantly improved, independent of product type (OR = 7.21, 95% CI 2.56, 20.34, *p* < 0.001, and OR = 2.82, 95% CI 1.50, 5.32, *p* = 0.001, respectively). The participants reported high satisfaction and acceptable taste, and no adverse effects were detected in those using the RSU gel throughout the study. Its lower cost and local availability make it a practical option for xerostomia management, particularly in populations with limited access to commercial saliva substitutes.

## 1. Introduction

Saliva plays an essential role in maintaining oral health by lubricating oral tissues and facilitating speech, taste, and mastication, as well as buffering and having antimicrobial effects [1]. Reduced salivary secretion or altered salivary composition is globally prevalent and can lead to xerostomia, oral discomfort, mucosal inflammation, increased susceptibility to infection, and a higher risk of dental caries, periodontitis, and candidiasis [2,3]. Xerostomia, the subjective feeling of dry mouth, and hyposalivation, a decrease in salivary flow, are oral conditions that significantly impair oral function and quality of life and are common in Thai patients [2,4,5]. The etiology of dry mouth is multifactorial, including aging, dehydration, psychological stress, autoimmune diseases such as Sjögren’s syndrome, head and neck radiotherapy, and, notably, medication-induced salivary gland hypofunction [5]. Common xerogenic medications include antidepressants, antihistamines, bronchodilators, and diuretics, all of which exert anticholinergic effects that reduce salivary flow [5,6].

Diagnosing dry mouth involves both subjective and objective assessments [7]. Questionnaires such as the Xerostomia Inventory (XI) and the Xerostomia-related Quality of Life scale (XeQoLs) assess the patient’s perceptions of dry mouth and its impact on quality of life, whereas sialometry objectively measures salivary flow [7]. Recently, devices such as the oral moisture meter (Mucus^®^) for determining oral wetness and the clinical score of oral dryness (CSOD) have been introduced as rapid, noninvasive tools for clinical assessment [8,9]. Managing dry mouth focuses on symptom relief, moisture replacement, and oral protection [10]; salivary stimulants and artificial saliva are the main therapeutic options [10]. Artificial saliva products, available in mouthwash, spray, or gel form, aim to mimic the lubricating properties of natural saliva [10,11]. However, commercial products available in Thailand, such as Biotène^®^ and GC Dry Mouth Gel^®^, are often costly and may not be affordable for long-term use, particularly for elderly or low-income patients. An oral moisturizing jelly developed in Thailand has high efficacy in patients with xerostomia following post-radiotherapy head and neck cancer or in elderly patients with hypertension and diabetes mellitus [12,13]. However, the inconvenience of transporting the edible jelly formulation in a box often reduces patient compliance.

Rangsit University (RSU) developed a cost-effective artificial saliva gel that provides moisture comparable to that of commercial products. Specifically, preliminary laboratory studies demonstrated that RSU formulations maintained a level of mucosal wetness equivalent to that achieved with GC Dry Mouth Gel^®^ [11]. However, the clinical benefits have not been elucidated. This study, therefore, aimed to evaluate the clinical effectiveness of the RSU artificial saliva mint gel compared with that of GC Dry Mouth Gel^®^, mint flavor, in patients with xerostomia. Changes in oral moisture, xerostomia symptoms, and clinical oral mucosal characteristics after single and continuous use for 14 days were investigated. Patient satisfaction and adverse events were also recorded. Findings of comparable effectiveness are expected to support the development of an affordable, practical alternative saliva substitute gel for xerostomia management in the Thai population.

## 2. Results and Discussion

### 2.1. Baseline Characteristics

Forty-one participants who were screened using the Thai version of the Summated Xerostomia Inventory (SXI) and 39 who met the inclusion criteria with an average SXI score of 8 (range: 6–13) were included in the study. Most of the participants (89.7%) were female, with an age range from 20 to 66 years. Eight (20.5%) participants had 1–4 medical problems, and seven (18.0%) of them were taking 1–6 drugs. There was no significant proportion of participants who smoked or drank alcohol. Two participants dropped out during the study, leaving thirty-seven who completed the short-term repeated-use study protocol (Table 1).

The range of SXI scores in our study was identical to that of Thai individuals with xerostomia enrolled in a validation study of the Thai version of the SXI [14]. Lapnimitanun et al. show that the patients’ Xerostomia Inventory (XI)-Thai and SXI-Thai scores demonstrated a strong correlation with each other and significant correlations with their scores on the standard xerostomia questions [14]. The Thai versions of the SXI and XI were reported to be valid, reliable, and easily administered instruments for assessing subjective dry mouth in Thai middle-aged and older individuals in both clinical and research settings [14]. Although our study population was similar to those in previous studies of patients with xerostomia [3,15,16], that most of our participants were younger, female, and without medical problems or medication use warranted a cautionary summary.

We therefore applied a crossover study design to strengthen the methodological rigor of the present clinical trial by minimizing inter-individual variability and enabling within-subject comparisons. The inclusion of a 14-day washout period between phases, based on similar crossover trials demonstrating the symptomatic relief provided by the saliva substitute gel, enabled the elimination of the gel’s lubricating effect on the newly formed oral mucosa, which is predictable according to the turnover rate of oral epithelium, effectively preventing potential carryover effects [17,18]. Researchers ensured that the oral tissue had fully regenerated and that any prior treatment had been eliminated, thereby making the results of the subsequent period more accurate [19]. We also analyzed the data using robust statistical methods appropriate for a crossover trial, including generalized linear mixed model (GLMM) and generalized estimating equation (GEE) adjusted for age and sex, to confirm that the product type, time, changes over time, evaluated period, and treatment sequence leading to a carryover effect did not significantly affect our outcomes, thereby validating the internal consistency and robustness of the findings. We expected that our results could be further generalized to the use of a saliva substitute gel in the general population.

The present study evaluated the core outcomes in objective and subjective aspects commonly used in dry mouth assessment [7]. Oral wetness, xerostomia severity score, CSOD, and patient satisfaction after product use, as recommended by the World Workshop on Oral Medicine (WWOM) VIII, were investigated to standardize our results and allow comparisons with other published data [7]. Adverse events from using the test gels were also determined to provide the best outcome for our product in future clinical settings.

### 2.2. Short-Term Analysis

**Figure 1:**
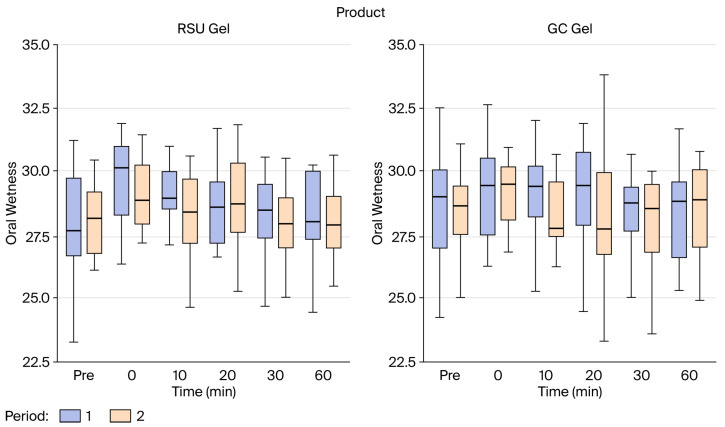
The oral wetness elicited with RSU and GC Dry Mouth Gel^®^ over 60 min.

**Figure 2:**
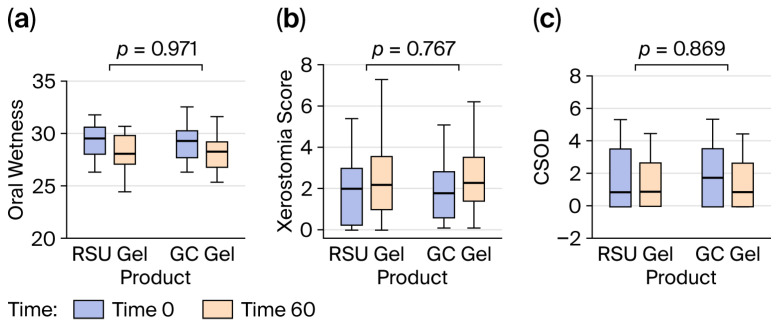
(**a**) The effects of product and changes over 60 min on oral wetness. (**b**) The effects of product and changes over 60 min on xerostomia score. (**c**) The effects of the product and changes over 60 min on CSOD value.

The oral wetness achieved with both gels measured over 60 min is depicted in Figure 1. Oral wetness elicited by the test (RSU gel) and control (GC Dry Mouth Gel^®^) gels increased immediately after application. It gradually decreased at 30 min, and at 60 min, oral wetness was significantly lower than at application time (29.2 to 28.2), a 3.34% change (95% CI 1.00, 5.70, *p* = 0.007). Differences between each treatment were indistinguishable. However, the mean wetness after both gel applications was higher (approximately 29.4 (95% CI 28.8, 29.9) and 29.1 (95% CI 28.5, 29.6) for the test and the control, respectively) than at the end of the period (approximately 28.4 for the test (F(1, 150) = 7.92, 95% CI 27.7, 29.0, *p* = 0.006) and 28.1 for the control (F(1, 150) = 7.60, 95%CI 27.4, 28.7, *p* = 0.007) product). Use of the RSU gel resulted in a mean unit reduction of 1.01, while use of the GC Dry Mouth Gel^®^ resulted in a mean unit reduction of 0.98. Neither the sequence of products applied nor the evaluation period affected the wetness achieved with either gel. The effects of the product and changes over 60 min on wetness (OR = 1.01, 95% CI 0.98, 1.04, *p* = 0.248; OR = 1.00, 95% CI 0.97, 1.04, *p* = 0.971) (Figure 2a), xerostomia severity score (OR = 1.10, 95% CI 0.42, 2.88, *p* = 0.661; OR = 1.15, 95% CI 0.45, 2.94, *p* = 0.767) (Figure 2b), and CSOD (OR = 0.95, 95% CI 0.58, 1.55, *p* = 0.765; OR = 0.94, 95%% CI 0.45, 1.95, *p* = 0.869) (Figure 2c) were, respectively, similar for the test relative to the control without a significant difference.

Our results align with previous reports of the transient moisturizing effects of saliva substitutes [10,17,18]. Both the RSU and GC Dry Mouth Gel^®^ elicited a marked, immediate increase in oral moisture, followed by a gradual decline within 60 min of application. Dry mouth relief from a saliva substitute is temporary because of the natural swallowing [10]. The duration of symptom relief with gel products has been reported as about 2 h [17,18]. These published data might also explain the similar xerostomia scores and CSOD values for both gels before and after 60 min of application [17,18]. In clinical practice, if patients wish to maintain xerostomia relief during the day, both products need to be applied more frequently than as specified in our study protocol [6]. Although frequent water sipping is a very cost-effective and easy-to-access method, using a saliva substitute gel is better for oral wetting, as it forms a protective film and mimics the lubricating properties of natural saliva for the oral mucosa [5,11,18]. The comparable oral wetness, xerostomia score, and CSOD values observed between the RSU and GC Dry Mouth Gel^®^ indicate that our gel provided comparable wetness, xerostomia relief, and changes in oral mucosal appearance as effectively as the well-known commercial product. This result supported our in vitro findings with the RSU gel showing that its wetting capacity was greater than that of water and lasted for at least 2 h [11].

### 2.3. Short-Term Repeated Use Analysis

#### 2.3.1. Oral Wetness

**Figure 3:**
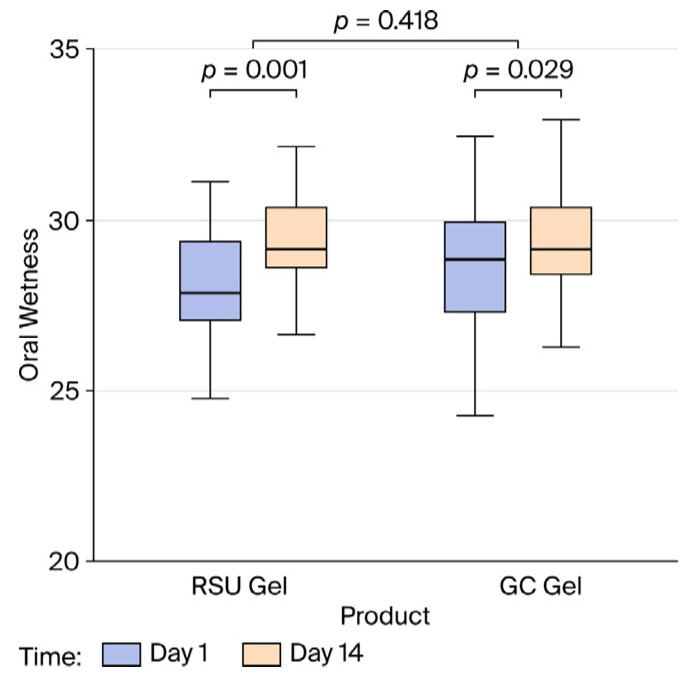
The effects of product and changes over 14 days on oral wetness elicited with RSU and GC Dry Mouth Gel^®^.

The product type did not affect the different levels of oral wetness achieved (*p* = 0.110). The change over time is also similar for both products (*p* = 0.418) and was not influenced by the sequence of products applied or the evaluated period. Furthermore, the overall wetness increased significantly from day 1 to day 14 (2.94%, 95% CI 0.30, 5.76, *p =* 0.030). The overall mean wetness on day 1 was approximately 28.3, while on day 14, it rose to around 29.4. This increase was statistically significant for both the test gel (a larger increase of 1.27, 95% CI 0.54, 2.01, *p* = 0.001) and the control gel (a difference of 0.85, 95% CI 0.09, 1.61, *p* = 0.029) (Figure 3). No difference in wetness was demonstrated between the products at the baseline or at the end of each period.

Since the study gel is designed to provide only symptomatic relief and contains no ingredients that stimulate or affect salivary flow rate, as similarly reported in previous studies [10,12,17,18,20,21], we therefore evaluated the oral mucosal wetness rather than the salivary flow rate. In addition, oral wetness is among the core outcomes for evaluating dry mouth, as recommended by the WWOM VIII [7]. The Mucus^®^ device for measuring oral moisture was developed by a group of Japanese researchers [8], who demonstrated a correlation between oral wetness measured by the device and subjective oral dryness, as well as objective measures of stimulated saliva [22]. The moisture level was also significantly different between the healthy group and the dry mouth group [8]. The intra-and inter-investigator reliabilities of the values measured using an oral moisture-checking device also aligned with those in published studies [23,24]. There was no significant influence of the operator on the oral mucosal moisture value measured with the device observed [24]. Although the pressure applied by the oral moisture-checking device influenced the measurement value, we supported the participant’s tongue during lingual mucosal moisture measurement to ensure uniform evaluation of oral moisture, as recommended in the literature [25]. Thus, the device could be helpful in a clinical setting for preliminary screening of dry mouth. However, the omission of sialometry in our study restricts the clarification of the correlation between subjective improvements and changes in salivary gland function. Future research should emphasize this limitation.

The continuous 14-day application of both gels four times daily led to sustained improvements in oral wetness. The RSU gel’s comparable clinical performance with that of the commercial gel may be attributed to its optimized formulation, which was previously demonstrated to provide a mucosal wetting capacity equivalent to that of GC Dry Mouth Gel^®^ in vitro [11]. A plausible explanation for this short-term repeated use increased wetness is that the distilled water with propylene glycol in the RSU gel serves as a moisturizer and humectant, and hydroxyethylcellulose thickens and forms a film, creating a protective hydration layer on oral mucosal surfaces [20]. All of these components provide moisturizing effects and confer mucosal-adherence properties. Previous studies have demonstrated that an oral moisturizing gel was most effective in enhancing unstimulated salivary flow rate compared to a mouthwash or spray, and the moisturizing gel’s effect on increasing saliva volume is related to the flavor of the gel, in addition to its moisturizing agent [5,26]. A sweet, minty, or acidic taste was more likely to increase saliva volume than a tasteless or odorless gel [26,27]. Our sweet mint-flavored saliva substitute gel was thus significantly more effective at increasing moisture in the short-term repeated use, whereas the short-term effect did not differ significantly from baseline.

#### 2.3.2. Xerostomia Score

**Figure 4:**
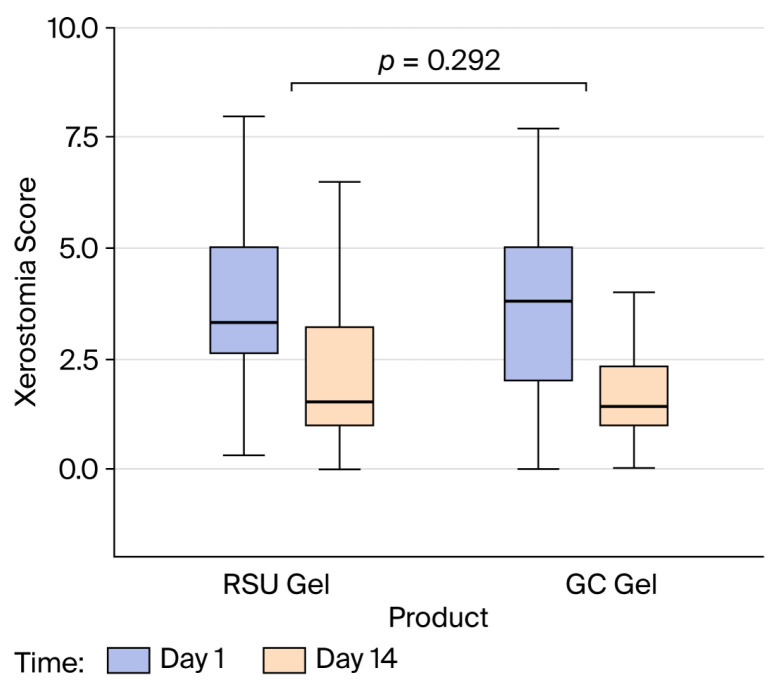
The effects of product and changes over 14 days on the xerostomia score elicited with RSU and GC Dry Mouth Gel^®^.

Neither the test nor the control gel (*p* = 0.292) nor the order of application (*p* = 0.740) had a significant impact on the xerostomia score after 14 days. The change in xerostomia scores over time was similar for both products, indicating that neither performed significantly better or worse than the other (*p* = 0.292) (Figure 4). The participants’ xerostomia significantly improved over the course of the study (*p* < 0.001), from the first to the second period (a 9.537-fold decrease in score). Nevertheless, the effect of the period of use of each product was also significant (*p* = 0.009), suggesting a significant difference in xerostomia scores between days 1 and 14, and days 29 and 43. Therefore, we further analyzed the xerostomia scores separately for each period. Interestingly, the xerostomia score decreased significantly, by 7-fold, in period 2 of the study (OR = 7.21, 95% CI 2.56, 20.34, *p* < 0.001), while no effect on the xerostomia score was significantly detected in period 1 (Wald 0.123, *p* = 0.726).

We evaluated the xerostomia severity according to the recommendations of the WWOM VIII consensus for assessing subjective dry mouth [7]. A 10 cm visual analogue scale was used to assess xerostomia severity before and after use of both experimental gels, ranging from 0 (no xerostomia at all) to 10 (most severe xerostomia) [28]. Because the treatment of xerostomia remains mainly symptomatic, it should therefore be evaluated using patients’ subjective scales, as in this study, since subjective impressions determine the burden for these patients [17]. Our study revealed a significant decrease in xerostomia after 14 days of use in the second period, in contrast to a previous study, which investigated a treatment over 7 days and found no significant effect or outcome on xerostomia relief [18]. Our results demonstrated that saliva substitute gels should be recommended to patients with xerostomia for long-term use, providing at least 2 weeks of comfortable oral moisture, as observed in a previous 28-day study [17]. Compared to a study in Thai elderly patients with hypertension and diabetes mellitus by Daludom et al., the use of an edible oral moisturizing jelly for 2 weeks significantly reduced dry mouth symptoms [13]. However, the participants’ potential learning effects, habituation to the questionnaire, or unintentional changes in behaviour during the second period of the current study must be taken into account when interpreting the significant results, though we used GEE statistics to control for the effect of the period. Long-term use spanning years and its effects on overall oral health and symptoms must be further researched.

#### 2.3.3. CSOD

**Figure 5:**
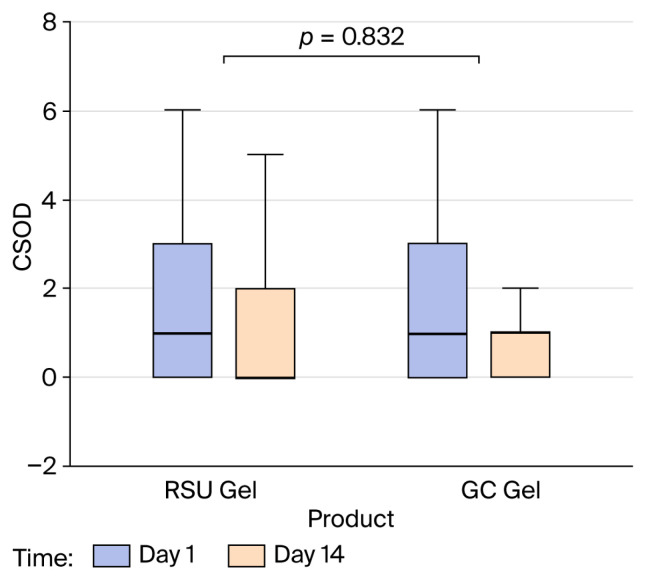
The effects of product and changes over 14 days on CSOD value elicited with RSU and GC Dry Mouth Gel^®^.

A similar trend to that of xerostomia was revealed in the CSOD assessment. Neither the test gel nor the control gel (*p* = 0.702) nor the sequence in which the products were administered (*p* = 0.525) had a significant effect on the participants’ oral mucosal status scores after 14 days. The change from day 1 to day 14 was consistent across the products (*p* = 0.832) (Figure 5). A significant decrease in CSOD values was observed between day 1 and day 14 (*p* = 0.006); however, the CSOD value differed between the two time periods (*p* = 0.012). A separate analysis was also conducted for each period, which showed that whereas there was no overall change in the CSOD between the two time points (days 1 and 14) in period 2 (*p* = 0.250), a significant difference in the CSOD between day 1 and day 14 was observed in period 1 (*p* = 0.002). As time progressed, the probability of a participant being in a lower CSOD category increased (OR = 2.82, 95% CI 1.50, 5.32, *p* = 0.001). At day 14, the test-gel group had a mean CSOD level of 0, whereas the control-gel group had a mean CSOD level of 1, which corresponded to the levels observed in healthy subjects in the previous study [9].

The mucositis index—determining erythema, ulceration, pain, swelling, inflammation, necrosis, and hemorrhage—did not change significantly in a study exploring two commercial saliva gel products over 28 days [17]. These parameters reflect the complications of long-term dry mouth; we therefore used the CSOD to evaluate early changes in the oral mucosa caused by dry mouth. The CSOD, recommended by WWOM VIII [9,29], was proposed as it is practical, easy to use, and reliable for routine clinical assessment of objective dry mouth severity; it not only evaluates the early signs of dry mouth but also examines its complications [9]. Osailan et al. demonstrated inverse relationships between the mean CSOD value and salivary flow rate and between the mean CSOD value and mucosal wetness [9]. Jager et al. also reported an association between CSOD value and unstimulated and stimulated salivary flow in a heterogeneous group of patients with hyposalivation, as well as a correlation between the CSOD and XI, the summated form of which we used to recruit our participants [30]. Based on the aforementioned data, we did not measure the salivary flow rate due to the short-term effect of the salivary substitute gel. We hypothesized that our novel saliva substitute gel could improve oral mucosal health through its wetting and lubricating properties, as reflected in clinically normal CSOD levels [9].

### 2.4. Response After Product Use

The consensus from WWOM VIII suggests exploring patients’ personal experiences with dry mouth, focusing on their perspectives on treatments and care outcomes [31]. A McNemar Test was performed to compare post-product-use response scores on day 14 across time points for each gel in the present study [18]. There was no statistically significant difference in the participants’ subjective response to the post-use questionnaire between the two products (*p* > 0.05) (Table 2). Almost all of the patients using the RSU gel answered “yes” to the dichotomous questions, “Did the product make your dry mouth better?” (97%), “Was the product easy to use?” (92%) and “Did you feel better for using this product?” (92%). The first three responses showed the same sequence as reported by Barbe et al. [18].

Additionally, four-fifths of the participants responded positively to the question “Did the product improve your quality of life?” Therefore, we conclude that the RSU product’s use did not result in a significant difference in satisfaction scores compared with the control gel, except for the need to continue using the product. Further review indicates that the younger participants with lower SXI scores were more likely to drive these neutral responses and intended to resume frequent water sipping for their symptom relief.

In addition to its stimulating effect on salivary flow, the gel’s mild, sweet–mint aroma might provide an enjoyable, refreshing taste for the participants with xerostomia, potentially influencing their willingness to use it [27,31]. The younger participants preferred the control gel’s stronger flavor and lower viscosity. In contrast, the elder participants reported that the test gel was more pleasant, more glutinous, and more long-lasting in their mouths compared to the younger participants. The elder participants expressed higher satisfaction with the RSU Gel’s texture, flavor, and mouthfeel—particularly its smooth consistency, less pungent mint flavor, and long-lasting lubricating effect—than the younger participants, which may have enhanced the older patients’ compliance with daily use. Texture and flavor are key factors that affect older adults’ food preferences [32]. A decrease in smell and taste perception is recognized as age-related, and sensory properties may directly influence their perceived measurements [33]. Despite the double-blind design and within-subject analyses used to minimize this potential bias, the distinct taste and texture differences between the gels could have biased our results, influencing subjective outcomes such as xerostomia scores and satisfaction ratings. Interpretation should therefore be made with caution. Nevertheless, our results are consistent with published data, indicating that a patient’s satisfaction with a product depends on more than just its active ingredients; a gel’s viscosity, flavor, visual appearance, and preparation form were identified as subjectively important to patients’ compliance with use [17,18,31,34,35].

Regarding these findings, it is interesting to note that patients are accustomed to strong-flavored products and consider them more effective, while sweet flavors are perceived as inappropriate for oral hygiene; therefore, the younger patients discontinued the test products because of their less minty flavor compared to the control gel, as observed from the only significantly different item in the questionnaire. Although the current study found lower positive responses to the questions on taste than to the other questionnaire items, over half of our participants reported reduced bad taste and improved taste sensation, findings similar to those reported in the literature [17]. Further efforts to improve patient compliance should be made to address these issues and increase acceptance of the test gel, making it more suitable for daily rather than occasional use.

According to our previous experiment, in addition to the RSU gel preparation, we developed a solution for application as a mouthwash or spray, yielding an oral wetness similar to that of a commercial saliva substitute mouthwash [11]. The RSU solution formula also provided oral wetness comparable to that of the RSU gel preparation at every time point. By 120 min, all of the saliva substitute gels and solutions were significantly more effective than water in maintaining mucosal wetness [11]. In a study of self-reported dry mouth symptoms, participants with xerostomia reported that mouthwash provided greater relief than water [21]. Mouthwashes containing lactoperoxidase, lysozyme, glucose oxidase, lactoferrin, or sodium hyaluronate, which are similar to components of naturally occurring saliva, provided better relief of xerostomia than those without [36,37]. Saliva substitute sprays, composed of trehalose or carboxymethylcellulose, were found to relieve oral dryness and improve xerostomia-related quality of life in Thai head and neck cancer patients with radiation-induced xerostomia [38]. We postulated that either our solution or a spray could serve as an alternative for patients with xerostomia, but future research is needed to confirm this assumption.

The latest systematic review and meta-analysis on the efficacy and safety of non-pharmacological interventions for xerostomia found that mouthwashes demonstrated the most significant improvement in xerostomia questionnaire scores (for assessing xerostomia severity, with scores scaled from 0 to 100) and XI scores (11 items rated on a 5-point Likert scale, where higher scores indicate greater symptom burden). At the same time, oral moisturizing gels showed the greatest reduction in visual analogue scale scores, which quantify perceived dry mouth severity on a 0–10 scale [39]. Therefore, saliva substitutes, such as mouthwashes and oral moisturizing gels, emerge as favorable first-line options for xerostomia relief [39]. Secondary benefits, such as the prevention of dental caries, gingivitis, or oral candidiasis, and individual patient preference, should guide product selection [13,17,18,31]. Future research on our test solution and gel should also explore behavioral, cultural, and logistical factors that influence patients’ compliance, as these determinants will be key to optimizing real-world treatment outcomes for patients with xerostomia.

Regarding the principal ingredients of the gels used in this study, distilled water with propylene glycol in the RSU gel or glycerin in GC Dry Mouth Gel^®^ serve as moisturizers and humectants. (Supplementary Tables S1 and S2) Although glycerin provides greater water-binding capacity, which is needed to keep the mouth moisturized for an extended period, propylene glycol was explicitly used to serve as a humectant, preservative, and antimicrobial ingredient to provide a preservative boost to ensure the gel remains safe and stable over time in our formula [11,18]. Due to its amphiphilic nature, propylene glycol acts as a mucosal permeation enhancer, facilitating deeper delivery of water into the cellular tissue and promoting sustained hydration [40]. In addition, propylene glycol offers lower viscosity and improved spreadability compared to glycerin, resulting in a lighter, more comfortable coating film. It also enhances peppermint flavor stability, thereby improving organoleptic properties (e.g., cooling sensation), which are critical for patient adherence [41]. This polyol creates a thermodynamic environment that prevents the recrystallization of other dissolved solutes, thereby maintaining the gel’s clarity and homogeneity. Propylene glycol’s multifunctional role is therefore highly useful in our specific oral formulation [11]. Hydroxyethylcellulose (HEC), a cellulose-based polymer, is used in both gels to form and thicken a film [20]. A robust hydrogen-bonding network forms between the aqueous molecules and the HEC, maintaining structural integrity further stabilized by the humectant system provided by propylene glycol. This dual-component system ensures the best performance of our RSU gel formulation. HEC does not undergo significant hydrolysis or ionization, which helps maintain the gel’s pH stability during extended storage [42]. Its non-ionic backbone maintains stable rheological properties even in the presence of salts such as calcium chloride, confirming the RSU gel’s long-term physicochemical stability [42]. All of the aforementioned ingredients confer moisturizing, lubricating, and mucosal-adherence properties to the gel. These ingredients might explain our similar findings of mucosal wetness and xerostomia relief with a commercial gel, as well as differences in preferences between the older and younger participants.

Xylitol was selected as a sweetener for our developed gel due to its antibacterial properties and lack of dental caries-causing effects [43]. It is possibly safe when used in chewing gums, candies, lozenges, toothpastes, and mouth products in amounts up to about 50 g daily, though it might cause allergic reactions, diarrhea, and flatulence in some people [44]. Sodium chloride, potassium chloride, and calcium chloride in the RSU gel, or sodium citrate in GC Dry Mouth Gel^®^, play primary roles in mimicking the electrolyte composition of natural saliva and providing buffering/pH stabilization. Nevertheless, calcium ions from calcium chloride in our novel gel facilitate enamel remineralization, thereby preventing dental caries, a significant complication of dry mouth. In contrast, sodium citrate simply helps saliva substitutes resist pH changes caused by acidic foods or bacterial activity, maintaining a near-neutral pH, which is critical for protecting tooth enamel from demineralization [45]. We therefore selected biocompatible, food-grade components that provide the most effective relief from dry mouth for our patients.

With the increasing number of patients with dry mouth, dentists need to be able to diagnose these problems, as patients often do not mention them, as we encountered before recruiting our participants [3,16,31]. Dentists must take a sufficient history from the patient, diagnose, and treat; they should inform these patients of the saliva substitute and which product they prefer. The cause of dry mouth should be corrected; if it cannot be changed, symptomatic management should be the next option, and attempts should be made to prevent complications of dry mouth [5,10,16]. The clinical effectiveness of our saliva substitute gel was confirmed, with no serious adverse events, and it was found to relieve patients’ dry mouth, suggesting it could be advised to improve their quality of life. The lower cost per 40-g tube of the RSU gel, THB 45 (≈USD 1.5), compared to that of GC Dry Mouth Gel^®^ (THB 480; ≈USD 16), is an optional advantage. More detailed and patient-centered questionnaires might have been more helpful in eliciting patient feedback on important factors influencing their long-term adherence to the products [31].

### 2.5. Amount of Gel Used

Both gel tubes were weighed before and after the 14-day application. The amounts of gel used were comparable between the two products: 12.5 g for the test gel and 12.2 g for the control gel.

Considering the same amount used for both studied gels, although lower than that reported by Barbe et al., similar results were obtained [18]. In clinical practice, the amount of gel used can vary according to the patient’s preference, as long as they feel comfortable using it for xerostomia relief [6]. Therefore, our product’s effectiveness was evaluated using subjective scales, as subjective impressions best reflect the burden among these patients, and objective measurements are yet to confirm the clinical effectiveness demonstrated. We believed that prescribing a greater amount of saliva substitute gel and advising a higher frequency of application would achieve a clinically meaningful moisture level, as observed in other studies [11,17]. Nevertheless, using the product with a high frequency would not be generalizable in general practice; therefore, future research on the amount of saliva substitute gel per use should clarify this assumption.

### 2.6. Adverse Events

Neither gel showed serious adverse events or intolerance, such as allergies, mucositis, or burning pain, as reported by participants or documented by the researchers, during or after use of the test gel. A slight burning sensation of the tongue from using a saliva substitute gel was reported in the study by Barbe et al., which resolved after a few minutes [18]. In their study, they prescribed a larger amount, around twice that in our study, of the investigated gels. This effect emphasizes the need for usage recommendations and an appointment after the first application. Sarideechaigul et al. developed artificial saliva formulations containing pilocarpine to treat xerostomia [46], and showed that their carboxymethylcellulose formulations helped relieve dry mouth symptoms by improving salivary flow rates. However, some adverse events, e.g., burning tongue, dizziness, and watery eyes, were reported [46]. Therefore, to avoid these adverse events and because of the topical effect of the saliva substitute gel, we did not add pilocarpine to our products. The frequency and amount per use and ingredients of the saliva substitute gel used should be tailored to patients’ preferences and the cause of their dry mouth. Nonetheless, if the products are used throughout the day, systemic absorption is likely to increase, and future research may need to evaluate potential side effects such as nausea or gastrointestinal disturbances. Further investigation using tools such as the Patient Assessment Questionnaire or the Patient-Reported Adverse Drug Event Questionnaire, in addition to open-ended questions and options for patients to indicate bothersome side effects, would provide more robust safety data, especially for detecting minor but relevant side effects [47].

We did not add preservatives such as ethylparaben, which were present in the commercial comparator gel, as they have been reported to cause adverse events; instead, we used potassium sorbate in our product. Ethylparaben is a highly effective, broad-spectrum synthetic preservative that works across a wide pH range but has raised significant public concern about its potential health effects, leading many brands to seek alternatives. Potassium sorbate is generally considered a safer, more naturally derived alternative that is highly effective against mold and yeast, as well as some bacteria, and has flavor-enhancing and antioxidant properties. It is also chemically compatible with the non-ionic nature of the HEC base, reinforcing long-term stability and ensuring a shelf-life that meets pharmaceutical standards for topical and oral applications, as well as for food and personal care products. Additionally, potassium sorbate is highly water-soluble, whereas ethylparaben is slightly soluble in water and more soluble in alcohol and oil-based solvents. We aimed to develop an alcohol-free saliva substitute gel, as a report has documented burning and tingling sensations associated with this ingredient, as well as from our clinical experience in treating patients with dry mouth [48]. Therefore, potassium sorbate was selected as our preservative because it is generally regarded as safe (GRAS) by the FDA for use in food [49].

## 3. Conclusions

The present study confirmed that both the novel RSU gel and the commercial GC Dry Mouth Gel^®^ provided similarly effective improvements in terms of oral wetness, xerostomia, and clinical oral dryness following regular use. There was no statistically significant difference between the two products, indicating comparable performance across sequences and application periods. The absence of serious adverse effects and the similarity in user satisfaction suggest that both gels are safe and well-tolerated. Consequently, the RSU gel demonstrates clinical equivalence to the commercial product, providing a lower-cost alternative for managing Thai dental patients experiencing xerostomia. Efficacy and adherence in older, medically compromised xerostomic patients, with more comorbidities or on more xerogenic medications, should be further investigated to confirm the generalizability of our results. Additionally, conducting randomized controlled trials over extended periods to evaluate the effects of this novel gel on clinical endpoints, such as the incidence of dental caries, periodontitis, and prevalence of oral candidiasis, would provide definitive evidence of its benefits.

## 4. Materials and Methods

### 4.1. Participants

Study participants were recruited from the Oral Diagnostic Clinic at Rangsit University’s College of Dental Medicine from July 2025 to September 2025. Dental patients aged 20 years or older were eligible for inclusion if their Thai SXI-5 score ranged from 6 to 15 at xerostomia screening, regardless of the underlying cause [14]. Exclusion criteria were patients with oral mucosal diseases or disease of the salivary glands; intake of other symptom-relieving products or artificial saliva besides water; actual head and neck tumor or radio- or chemotherapy in this area; any known allergy to the test or control products; and actual or planned pregnancy. All eligible and willing participants were invited to participate in the study after signing a written informed consent form.

### 4.2. Sample Size Calculation

The sample size was calculated based on our previous study on mucosal wetness elicited by the test product [11]. We expected a mean difference in wetness of 2 and a standard deviation (SD) = 2. In a crossover design with a positive correlation, *p* = 0.05, α = 0.05, and power (1 − β) = 0.8, a sample size of 32 subjects is required, assuming a dropout rate of 10%, resulting in 36 subjects (18 per group).

### 4.3. Study Design

This double-blinded randomized crossover trial was approved by the Ethics Review Board of Rangsit University and conducted in full accordance with the Declaration of Helsinki (COA No. RSU–ERB2025–123) and was also registered with the Thai Clinical Trials Registry (registration number TCTR20250309006; https://www.thaiclinicaltrials.org/) (accessed on 9 March 2025) before the first patient was enrolled. The present study was conducted in two parts: a single-use assessment of the researched products’ short-term effectiveness and a repeated-use assessment of both products over 14 days, with a 14-day washout period (short-term repeated use effectiveness).

#### 4.3.1. Short-Term Study

On the first day, a clinical oral examination, medical history-taking, and medication review were conducted. All participants were instructed to refrain from eating, drinking, or tooth brushing for 1 h prior to data collection. Participants were randomized to treatment with the test product (RSU gel) or the control product (GC Dry Mouth Gel^®^; GC Corporation, Tokyo, Japan). Every participant applied the first mouth gel, which was followed by evaluation of oral wetness, completion of the xerostomia questionnaire, and examination of CSOD; this was repeated at 10, 20, 30 and 60 min. After a 60-min washout period (with water), the second mouth gel was applied. At the abovementioned time, the parameters were assessed again.

#### 4.3.2. Short-Term Repeated Use Study

**Figure 6:**
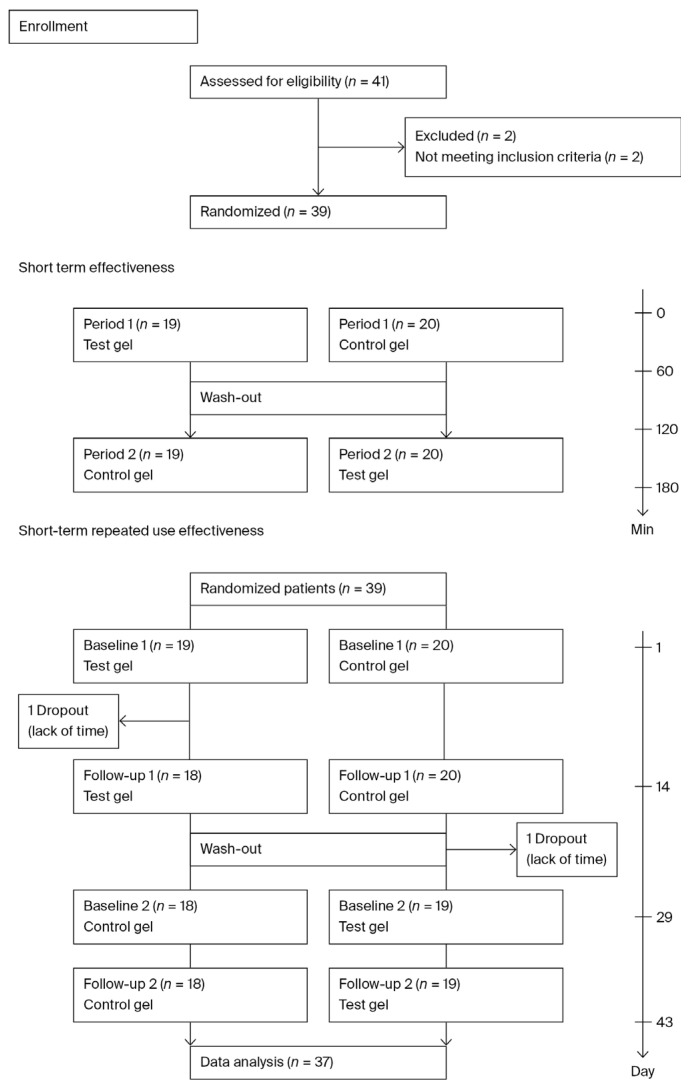
CONSORT study flow chart.

Following the short-term study, all participants were assigned to receive either the test or control gel for a 14-day treatment period (Period 1). Following period 1, a 14-day washout period was observed, followed by period 2 (day 29–43), during which participants crossed over to the second product for a second 14-day application. The first application of each product was performed by the participants themselves under the recommendation of the researcher dentists, who provided both oral and written instructions. Participants were instructed to apply 1 inch of the gel squeezed onto marked wooden sticks to the tongue dorsum, then swish it around the mouth three times after a meal and before bedtime each day. During the washout period, participants had to refrain from using the study products or any other xerostomia-relieving products. Tooth brushing and regular toothpaste, except mouthwash, could be used as usual (Figure 6).

### 4.4. Blinding and Randomization

Participants and clinical investigators were blinded regarding the products. Randomization of the products was performed by a researcher (WK) who was not involved in the clinical trial via the NIH Clinical Trial Randomization Tool (https://ctrandomization.cancer.gov/tool/) (accessed on 2 June 2025). The program provided an allocation sequence for each product, and a dental assistant not involved in the study handed out the unlabeled products to the participating dentists in the order specified.

### 4.5. Salivary Test and Control Gels

GC Dry Mouth Gel^®^ is a sugar-free, transparent gel that provides oral comfort and lubrication by moistening the mouth, tongue, and throat. It is a commonly used saliva substitute gel commercial product available over the counter and online in Thailand. Its ingredients include glycerin, aqua, cellulose gum, carrageenan, sodium citrate, aroma (mint flavor was selected for this research), ethylparaben, and benzyl alcohol. Its neutral pH helps maintain oral pH within a safe range, preventing demineralization.

RSU mint gel was produced from materials ordinarily used in oral cosmetics or foods, approved by the FDA, and that have demonstrated effective wetness capacity in an in vitro study [11] (Table S2). A formulation aimed not only to moisten the oral mucosa but also to replicate the complex rheological, buffering, and protective hydration layer properties of natural saliva. Its ingredients include propylene glycol, hydroxyethylcellulose, distilled water, essential chloride-based electrolytes, xylitol, potassium sorbate, and peppermint oil. The advantage of the RSU gel is that it does not contain ethylparaben, hydroxybenzoate, or alcohol, which are usually the cause of adverse reactions [11,18,46,48].

RSU mint gel was prepared via controlled-temperature, high-shear homogenization to achieve structural uniformity and optimal rheology. The HEC matrix was initially hydrated in distilled water (pH 7.0) under constant mechanical agitation (500–800 rpm) at room temperature. To maintain polymer stability and prevent electrolyte-induced collapse, a secondary aqueous-glycolic phase—comprising sodium chloride, calcium chloride, potassium chloride, xylitol, and potassium sorbate—was incorporated incrementally via slow titration. During this stage, the temperature was progressively increased. Peppermint oil was incorporated at 45 °C and stabilized with pharmaceutical-grade polyols to ensure uniform dispersion and prevent phase separation. The final mixture was heated to 50 °C and stirred until a transparent, homogeneous gel was obtained. The viscoelastic properties of the synthesized gel were quantitatively characterized using a calibrated Brookfield™ rotational viscometer (AMETEK Brookfield, Middleborough, MA, USA). To evaluate flow behavior and structural integrity, measurements were conducted at 25 °C. A range of shear rates was applied by varying the spindle speed to determine the dynamic viscosity and identify non-Newtonian behavior, such as shear-thinning or thixotropy. The torque range was maintained between 10% and 100% to ensure measurement accuracy in accordance with the manufacturer’s calibration standards. The acidity/alkalinity of the gel matrix was monitored using a high-precision digital pH meter (Mettler Toledo, Columbus, OH, USA) equipped with a glass electrode. Prior to measurement, the device underwent a three-point calibration using standard NIST-traceable buffer solutions (pH 4.01, 7.00, and 10.01). The electrode was stabilized within the gel matrix until a constant reading (drift < 0.01 units/min) was achieved, ensuring the precision of the electrochemical potential. The RSU gel’s viscosity of 170,000 ± 14,250 centipoise at room temperature and pH 7.0 ± 0.05 was finally retrieved.

Both the test product (RSU gel) and the control product (GC Dry Mouth Gel^®^) tubes were provided by a researcher (WK) who was not involved in the clinical study phase. All tubes were weighed at 40 g per tube before being provided to participants. Both products were packed identically without any identifying features and stored with a person not otherwise involved in the study. Each participant received one tube of the relevant gel at each start of the respective short-term repeated-use trial. At the end of the 14-day application period, participants returned the gel tubes, and all of them were weighed again.

### 4.6. Parameter Assessed

Age, sex, medical history, medication use, allergies, smoking, and drinking habits were evaluated and recorded.

#### 4.6.1. Oral Wetness Measurement

A Digital Moisture Analyzer/Oral Moisture Checking Device (Mucus^®^, product number: 441244; Life Co., Ltd., Saitama, Japan), developed by a group of Japanese researchers, was used to measure oral wetness according to the manufacturer’s instructions [8]. The device sensor was covered with a sterile polyethylene disposable bag (thickness, 12 µm) and pressed to the tongue mucosa at 200 g of pressure until a beeping sound was heard (approximately 3 s). Oral wetness was measured three times on the middle third of the dorsum of the tongue, where each formula of saliva gel was applied, and the median value was used for analysis, as per the manufacturer’s instructions. Regarding the interpretation of oral dryness in individuals, values ≥ 29.6% are considered normal, 28.0–29.5% are borderline, and ≤27.9% are low [8]. The oral wetness measurement intervals for each product were pre-application (pre); baseline (i.e., immediately after application of the experimental saliva gels; time: 0 min); and at 10, 20, 30, and 60 min after application, including at day 1 and day 14 of each period.

#### 4.6.2. Clinical Score of Oral Dryness (CSOD)

The CSOD was assessed by the principal investigator (ST) after oral wetness was measured (Figure S1). Ten objective clinical features were identified as being reliable for routine assessment of dry mouth severity, and an inverse relationship was reported between CSOD and both mucosal wetness and salivary flow rate [9].

#### 4.6.3. Questionnaires

All participants completed questionnaires in a quiet room. Participants could take as much time as they needed without interference. The xerostomia questionnaire included a 10 cm visual analogue scale to assess xerostomia severity, ranging from 0 (no xerostomia at all) to 10 (most severe xerostomia) [7,17,28]. The participants were asked to complete the questionnaire at the beginning and end of each period.

The subjective after-product-use questionnaire was also completed after every 14 days of product use [18]. The questions comprised dichotomous yes/no response variables that investigate effectiveness, satisfaction with taste improvements, quality of life, and improvements in xerostomia-related aspects of daily life [18]. Additionally, participants were asked verbally whether they experienced any adverse events or side effects and their responses were recorded.

### 4.7. Statistical Analysis

Normality was assessed using the Shapiro–Wilk test. Data were evaluated descriptively; frequencies and percentages were calculated for qualitative variables, and the median and interquartile range, or the mean and SD, were reported for quantitative variables, as appropriate.

Regarding the crossover design for repeated measurements, a GLMM was used to evaluate normally distributed oral wetness, with product, time, period, sequence, and the interaction of product and time (i.e., changes over time) as fixed effects to control potential confounding effects (period and carryover/sequence effects) with significance set at *p <* 0.05. Generalized estimating equations were used to analyze the non-normal distributions of ordinal scores for xerostomia and CSOD and to estimate equivalent main effects, as observed in the analysis of oral wetness.

Short-term effects were analyzed across multiple time points (0–60 min), and short-term repeated use effects were compared between day 1 and day 14 for both products, while also assessing product order and carryover effects. Descriptive and ratio analyses were performed to ensure group comparability, and results were expressed as estimated means and SDs, along with significance values as odds ratios (ORs) and 95% confidence intervals (CIs), obtained from the respective models. A McNemar Test was performed to compare satisfaction scores. All reported *p*-values at 0.05 are considered statistically significant. All analyses were performed with SPSS Statistics 27 (IBM Corp., Armonk, NY, USA).

## Figures and Tables

**Table 1:** Baseline clinical characteristics of the participants.

	Period 1: RSU Gel Period 2: GC Dry Mouth Gel ^®^ (*n* = 19)	Period 1: GC Dry Mouth Gel^®^ Period 2: RSU Gel (*n* = 20)	All (*n* = 39)
	*Median* *(1st, 3rd quartile)*	*Median* *(1st, 3rd quartile)*	*Median* *(1st, 3rd quartile)*
Age (years)	43 (23, 57)	24 (21, 50)	24 (21, 52)
SXI	8 (7, 10)	8 (7, 9)	8 (7, 9)
	*Count (%)*	*Count (%)*	*Count (%)*
Sex			
Male	2 (10.5)	2 (10.0)	4 (10.3)
Female	17 (89.5)	18 (90.0)	35 (89.7)
Medical problems Yes	5 (26.3)	3 (15.0)	8 (20.5)
Medication use Yes	5 (26.3)	2 (10.0)	7 (18.0)

SXI: Summated Xerostomia Inventory.

**Table 2:** Post-product-use response to the test and control gel on day 14 of use of the respective product. The values shown are for those participants who responded “yes”; * McNemar Test.

	Test *n* (%)	Control *n* (%)	p *
Did the product make your dry mouth better?	36 (97)	36 (97)	1.000
Did the product make chewing easier?	29 (78)	27 (73)	1.000
Did the product make swallowing easier?	29 (78)	31 (84)	0.687
Did the product make talking easier?	30 (81)	30 (81)	0.687
Did the product improve your sensation of taste?	22 (60)	22 (60)	1.000
Did the product reduce any bad taste that you get in your mouth?	21 (57)	23 (62)	0.388
Did the product stop you from waking in the night?	24 (65)	27 (73)	0.549
Would you like to continue using this product?	25 (68)	29 (78)	0.006
Did the product improve your quality of life?	31 (84)	34 (92)	0.063
Did you speak to people more than you used to?	25 (68)	23 (62)	1.000
Did you get out of the house more than you used to?	20 (54)	21 (57)	1.000
Was the product easy to use?	34 (92)	35 (95)	1.000
Did you feel better for using this product?	34 (92)	34 (92)	0.125
If you have a burning mouth, did the product improve the burning sensation?	3 (8)	4 (11)	1.000
Was the product most useful in the day or at night?	Day: 21 (57) Night: 16 (43)	Day: 21 (57) Night: 16 (43)	0.289

## Data Availability

The data supporting the reported results can be obtained from the corresponding author.

## Supplementary Materials

The following supporting information can be downloaded at https://www.mdpi.com/article/10.3390/gels12010061/s1. Table S1: Comparison of the ingredients between RSU and GC Dry mouth gel^®^; Table S2: Percentage of RSU gel formulation (*w*/*w*); Figure S1: Clinical score of oral dryness (CSOD).

## Abbreviations

The following abbreviations are used in this manuscript:

XI	Xerostomia Inventory
XeQoL	Xerostomia-Related Quality of Life scale
CSOD	Clinical Score of Oral Dryness
RSU	Rangsit University
SXI	Summated Xerostomia Inventory
GLMM	Generalized Linear Mixed Model
GEE	Generalized Estimating Equation
WWOM	World Workshop on Oral Medicine
NIH	National Institutes of Health
FDA	Food and Drug Administration
SD	Standard Deviation
OR	Odds Ratio
CI	Confidence Interval
