# Supplementary material for: Comparable Effectiveness of Novel and Commercial Saliva Substitute Gels in Dental Patients Experiencing Xerostomia: A Randomized, Double-Blind Crossover Trial
Source: Gels. 2026 Jan 8;12(1):61. doi: 10.3390/gels12010061 (PMC12840857; doi:10.3390/gels12010061)
Supplement: Supplementary file 1 [file gels-12-00061-s001.zip › Figure S1 - CSOD Manual - Supanee Thanakun.pdf]

Clinical score oral dryness (CSOD)

Item	Oral examination	Clinical characteristics
1.	Mouth mirror sticks to buccal mucosa	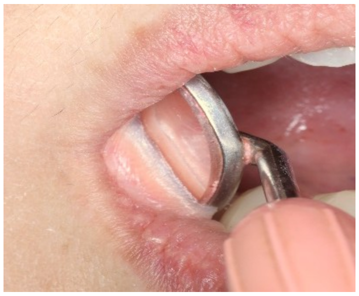
2.	Mouth mirror sticks to tongue	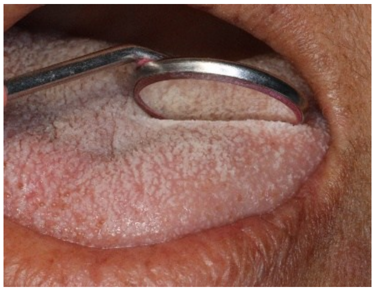
3.	Frothy saliva	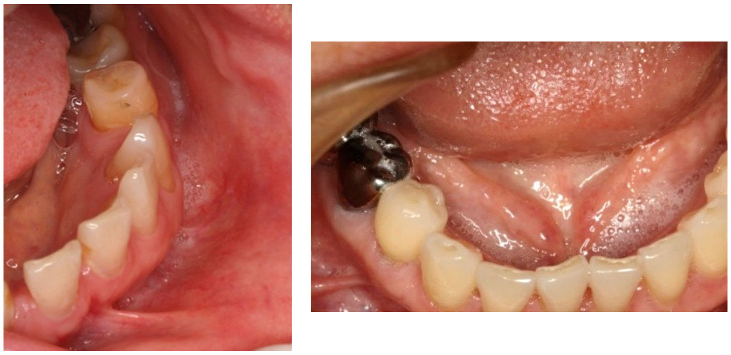
4.	No saliva pooling in floor of mouth	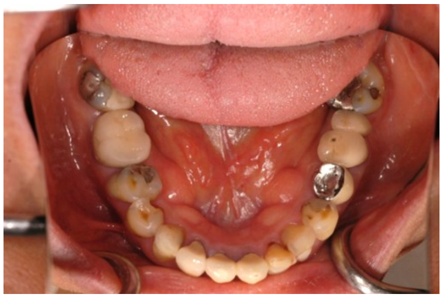
5.	Tongue depapillation	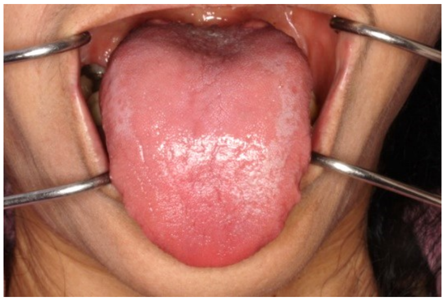

Clinical score oral dryness (CSOD)

Item	Oral examination	Clinical characteristics
6.	Altered gingival architecture (i.e. smooth)	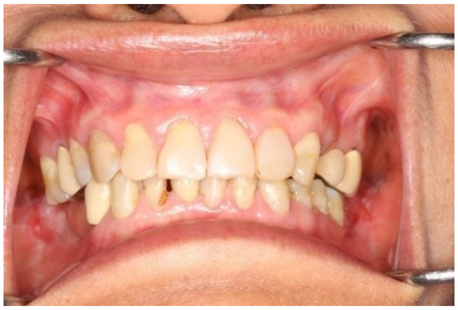
7.	Glassy appearance of oral mucosa, especially palate	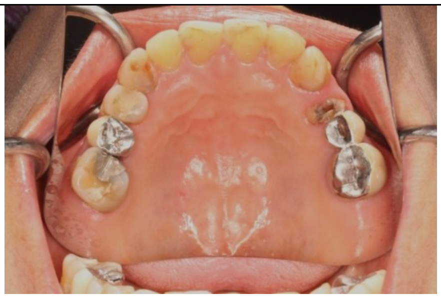
8.	Lobulated/fissured tongue	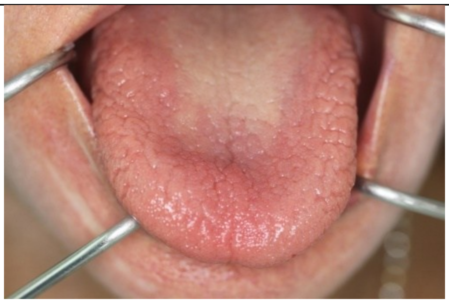
9.	Cervical caries (> 2 teeth)	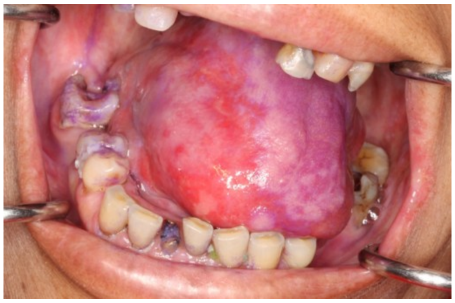
10.	Debris on palate or sticking to teeth	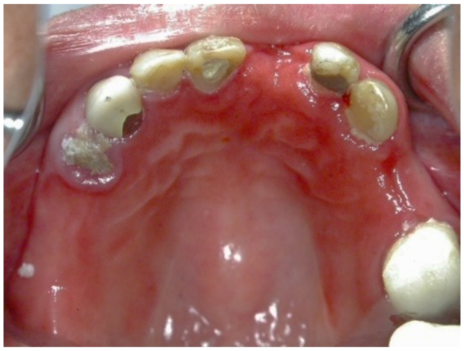
