# Supplementary material for: Comparable Effectiveness of Novel and Commercial Saliva Substitute Gels in Dental Patients Experiencing Xerostomia: A Randomized, Double-Blind Crossover Trial
Source: Gels. 2026 Jan 8;12(1):61. doi: 10.3390/gels12010061 (PMC12840857; doi:10.3390/gels12010061)
Supplement: Supplementary file 1 [file gels-12-00061-s001.zip › Table S1 - Supplementary data - RSU and GC saliva substitute gel - Ingredients.pdf]

Table S1 Comparison of the ingredients between RSU and GC Dry mouth gel®

Saliva substitute ingredients	Proposed functions	RSU Gel	GC Dry mouth Gel®*
Distilled water	Formulation solvent basis	O	O
Propylene glycol	Moisturizer/Humectant/Solvent	O	
Glycerin	Moisturizer/Humectant		O
Hydroxyethyl cellulose	Thickener	O	O
Carrageenan	Thickener, gelling agent		O
Sodium chloride<br>Potassium chloride<br>Calcium chloride	Buffer	O	
Sodium citrate	Buffer		O
Xylitol	Sweetener,<br>Antibacterial agent	O	
Potassium sorbate	Preservative	O	
Ethylparaben	Preservative		O
Peppermint oil	Aroma	O	
Flavor	Aroma		O

* https://www.westterracedental.co.uk/product/gc-dry-mouth/#tab-additional_information
