# Supplementary material for: Comparable Effectiveness of Novel and Commercial Saliva Substitute Gels in Dental Patients Experiencing Xerostomia: A Randomized, Double-Blind Crossover Trial
Source: Gels. 2026 Jan 8;12(1):61. doi: 10.3390/gels12010061 (PMC12840857; doi:10.3390/gels12010061)
Supplement: Supplementary file 1 [file gels-12-00061-s001.zip › Table S2 - Supplementary data - RSU gel - Ingredients.pdf]

Table S2 Percentage of RSU gel formulation (w/w)

Saliva substitute ingredients	Percentage of RSU gel formulation (w/w)	Functions
Propylene glycol	5 - 15	Humectants, Preservatives
Hydroxyethylcellulose	1 - 3	Viscosity-increasing agent
Sodium chloride	0.01 - 0.1	Ion concentration adjusting agent
Potassium chloride	0.01 - 0.1	Ion concentration adjusting agent
Calcium chloride	0.01 - 0.1	Ion concentration adjusting agent
Xylitol	2 - 5	Sweetener
Potassium sorbate	0.025 - 0.1	Preservatives
Peppermint oil	0.05	Flavoring agents
Distilled water	qs. to 100	Formulation solvent basis
